# Patient-Reported Concerns and Factors Associated With Anesthetic Preference: A Cross-Sectional Study on Why Patients Choose General Over Regional Anesthesia

**DOI:** 10.7759/cureus.117201

**Published:** 2026-09-30

**Authors:** Paraskevi Mavridou, Pinelopi Kitsakou, Panagiota Panagiotou, Christos Exarchos, Chrysoula Antigoni Koutsi, Athina Stratsiani, Marina Gkegka

**Affiliations:** 1 Department of Anesthesiology, General Hospital of Ioannina "G. Hatzikosta", Ioannina, GRC

**Keywords:** anesthetic preference, general anesthesia, patient concerns, preoperative care, regional anesthesia, shared decision-making

## Abstract

Background

Regional anesthesia (RA) offers established perioperative benefits, yet many patients choose general anesthesia (GA) even when RA is a clinically appropriate option. Understanding patients' specific concerns and other factors associated with anesthetic preference may help improve patient-centered preoperative communication and shared decision-making.

Methods

This cross-sectional, questionnaire-based study included 276 adult patients scheduled for elective surgery at a public general hospital in Greece. Before preoperative anesthetic consultation, participants reported their anesthetic preference, previous RA experience, and sociodemographic and clinical characteristics. Patients who preferred GA identified their concerns regarding RA from 11 predefined items. Factors independently associated with anesthetic preference were assessed using multivariable binary logistic regression.

Results

Of 350 eligible patients invited, 276 completed the survey (response rate = 78.9%); 65.9% (n = 182) preferred GA and 34.1% (n = 94) preferred RA. Male sex (odds ratio {OR} = 7.12; 95% confidence interval {CI}: 3.13-16.22; p < 0.001) and previous RA experience (OR = 3.50; 95% CI: 1.70-7.19; p = 0.001) were independently associated with RA preference. Surgical specialty, education, and employment status were also independently associated with anesthetic preference. Among patients preferring GA (n = 182), the most frequently reported concerns were hearing events during surgery (90.1%, n = 164), seeing the procedure (77.5%, n = 141), and experiencing intraoperative pain (73.1%, n = 133). Concerns about permanent paralysis (40.7%, n = 74) and back injury (40.1%, n = 73) were less frequent. Among respondents to the information-related items (n = 202), 31.2% (n = 63) reported having heard that RA could cause complications, most commonly from relatives (66.7%, n = 42), while 95.5% (n = 193) wanted their anesthesiologist to address their questions about RA.

Conclusions

GA was preferred by approximately two-thirds of elective surgical patients despite RA being a clinically viable option. The leading concerns regarding RA centered on the awareness of the surgical environment and intraoperative pain rather than rare neurological complications. Anesthetic preference was also independently associated with previous RA experience and several patient characteristics. Identifying and directly addressing these concerns during preoperative counseling may support more informed and individualized anesthetic decision-making.

## Introduction

Regional anesthesia (RA), including neuraxial techniques and peripheral nerve blocks, is an established component of modern perioperative care. It can provide effective analgesia, reduce systemic opioid requirements, avoid some adverse effects associated with general anesthesia (GA), and facilitate postoperative recovery [1]. Despite these advantages, many patients choose GA even when RA represents a clinically appropriate option [2,3].

Anesthetic preference is influenced not only by the perceived benefits and risks of each technique but also by patients' individual concerns, previous experiences, and understanding of anesthesia. Fear of needle insertion, pain during block placement or surgery, remaining awake, seeing or hearing events during the procedure, back injury, neurological injury, and paralysis have all been reported as reasons for reluctance to undergo RA [3,4]. Previous anesthetic experience may also influence subsequent preference [3], while limited knowledge and misconceptions regarding RA remain common [5,6].

Effective preoperative communication may therefore play an important role in anesthetic decision-making. However, providing general information about the risks and benefits of different anesthetic techniques may not fully address the specific concerns that influence an individual patient's choice. Recent evidence has also emphasized the importance of patient involvement and shared decision-making in the selection of anesthetic technique [7]. In particular, it remains unclear whether concerns related to the immediate sensory experience of surgery while conscious, such as hearing or seeing events in the operating room or experiencing pain, are more prominent in patients' preferences than concerns about rare but serious complications of RA.

The present study aimed to evaluate anesthetic preferences among adult elective surgical patients for whom RA was a clinically viable option. It also sought to identify patient-reported concerns associated with choosing GA over RA and to determine the sociodemographic and clinical factors independently associated with anesthetic preference. Additionally, we explored patients' sources of information regarding RA and their desire to have their questions addressed by an anesthesiologist.

Preliminary findings from this study were previously presented as an e-poster at Euroanaesthesia 2026, Rotterdam, the Netherlands, on June 6-8, 2026.

## Materials and methods

Study design and setting

This cross-sectional, questionnaire-based study was conducted at a public general hospital in Greece serving a regional population of approximately 520,000. Data collection was performed from April to June 2025 following ethical approval and pilot testing. Consecutive adult patients scheduled for elective surgery were screened for eligibility.

Patients were eligible if they were aged ≥18 years and scheduled for an elective procedure for which regional anesthesia (RA), including neuraxial or peripheral nerve block techniques, represented a clinically viable anesthetic option. The clinical viability of RA was determined according to the type of surgical procedure and the department's usual anesthetic practice and was confirmed by an anesthesiologist for each patient. Participants were required to be able to understand Greek and independently complete the questionnaire.

Patients undergoing emergency or urgent surgery were excluded, as were those with cognitive impairment, acute delirium, severe psychiatric or neurological disorders affecting communication, inability to read or understand Greek, or sensory or physical limitations preventing independent questionnaire completion. Patients with American Society of Anesthesiologists (ASA) physical status IV or requiring immediate postoperative intensive care were also excluded. Questionnaires with incomplete or illegible responses to the required core items were excluded from the analysis.

Sample size and sampling

The minimum required sample size was estimated using Cochran's formula for proportions: n₀ = Z²p(1-p)/e², where n₀ is the required sample size, Z is the standard normal value corresponding to the desired confidence level, p is the expected population proportion, and e is the desired margin of error [8]. Using a 95% confidence level (Z = 1.96), a 6% margin of error (e = 0.06), and an expected proportion of 50% (p = 0.50), the calculation was n₀ = (1.96² × 0.50 × 0.50)/(0.06²) = 266.78, which was rounded up to 267 participants. To allow for nonresponse and incomplete questionnaires, 350 eligible patients were approached. Consecutive sampling was used throughout the study period. Of the 350 patients approached, 276 provided completed questionnaires and were included in the final analysis, resulting in a response rate of 78.9%.

Questionnaire development and pilot testing

A study-specific questionnaire was developed by the investigators after reviewing the relevant literature on patients' perceptions, fears, and concerns regarding regional anesthesia [3,4]. The previously published studies were used to inform the content and domains considered relevant to the present study; the questionnaire used in this study was developed by the investigators specifically for the study population and setting and was not a reproduction or translation of either previously published questionnaire. The questionnaire was developed in Greek using simple clinical terminology to ensure comprehensibility across different educational levels.

A panel of senior anesthesiologists reviewed the questionnaire for clinical relevance, clarity, and content. The final questionnaire included 11 predefined concerns considered relevant to the study population, with no free-text option for reporting additional concerns. The preliminary version was pilot-tested in March 2025 in 20 elective surgical patients of different ages, sexes, and educational backgrounds. Participants were subsequently interviewed regarding the clarity and comprehensibility of the questionnaire, and minor wording modifications were made where necessary. Pilot participants were not included in the final analysis.

The complete study-specific questionnaire administered to participants, including the original Greek version and its English translation, is provided in the Appendices.

Data collection

Because the hospital does not operate an outpatient preoperative anesthesia clinic, questionnaires were distributed on the surgical ward on the day before surgery and completed before the scheduled preoperative anesthetic consultation. Completed questionnaires were collected by a designated researcher before the anesthesiologist's visit to capture patients' baseline preferences and concerns before individualized professional counseling.

Before indicating their anesthetic preference, participants received a brief standardized written explanation of GA and RA as part of the questionnaire. RA was described as spinal or epidural anesthesia or a peripheral nerve block, involving local anesthetic administration to produce numbness and allow surgery without pain and without the need for deep sleep. The explanation also stated that medication for light sedation could be administered if desired.

The questionnaire collected sociodemographic and clinical characteristics, previous RA experience, and preferred anesthetic technique. Patients who preferred GA were asked to report their concerns regarding RA using a predefined list of 11 items. Questions concerning previous information about potential complications of RA, the source of this information, and the desire for further information from the anesthesiologist were positioned within the section intended primarily for participants who did not prefer RA. These items were not mandatory for the entire study population; however, some participants who ultimately indicated a preference for RA also completed them. Analyses of these information-related items were therefore based on the available responses, with the corresponding denominator reported for each analysis; no imputation of missing responses was performed.

Ethical considerations

The study protocol was reviewed and approved by the Scientific Council of General Hospital of Ioannina "G. Hatzikosta" (approval number: 1/28-01-2025) at Meeting Number 1 on January 28, 2025 (Subject 7, Study 1). The study was conducted in accordance with Good Clinical Practice and applicable ethical principles. Before questionnaire administration, patients were informed in writing about the purpose of the study and the voluntary and anonymous nature of participation. The completion and return of the questionnaire indicated their agreement to participate.

Statistical analysis

Statistical analysis was performed using IBM SPSS Statistics for Windows version 26.0 (IBM Corp., Armonk, NY). Continuous variables were summarized using appropriate measures of central tendency and dispersion, while categorical variables were presented as frequencies and percentages.

Associations between categorical variables and anesthetic preference were assessed using Pearson's chi-square test. Age was compared between preference groups using the Mann-Whitney U test.

Multivariable binary logistic regression was performed to identify factors independently associated with anesthetic preference. Variables showing statistically significant associations with anesthetic preference in the bivariate analyses were included in the multivariable model; these were sex, age, surgical specialty, educational level, previous RA experience, and employment status. Results are presented as odds ratios (ORs) with 95% confidence intervals (CIs). Multicollinearity was assessed using variance inflation factors (VIFs), with all values below 5. Linearity in the logit for age was assessed using the Box-Tidwell approach and showed no statistically significant departure from linearity (p = 0.086). Model calibration was assessed using the Hosmer-Lemeshow goodness-of-fit test. A two-sided p-value of <0.05 was considered statistically significant.

## Results

Characteristics of the study population and anesthetic preference

Of 350 eligible patients invited to participate, 276 completed the questionnaire, corresponding to a response rate of 78.9%. The median age was 67.5 years (Interquartile range {IQR} = 23.0; range: 20-88), and 58.0% (n = 160) of participants were men. Most participants had previously undergone anesthesia (82.2%, n = 227), while 35.5% (n = 98) had previous experience with RA. The demographic and clinical characteristics of the study population are presented in Table 1.

**Table 1:** Demographic and clinical characteristics of the study population (N = 276). Values are reported as counts (n) and percentages (%) unless otherwise indicated. Age is reported as median (IQR) and range. IQR: interquartile range

Characteristics	n (%)
Age, years	
Median (IQR)	67.5 (23.0)
Range	20-88
Sex	
Male	160 (58.0)
Female	116 (42.0)
Surgical specialty	
Orthopedics	99 (35.9)
General surgery	61 (22.1)
Urology	85 (30.8)
Obstetrics/gynecology	31 (11.2)
Educational level	
Primary education	97 (35.1)
Secondary education	68 (24.6)
High school	41 (14.9)
Higher/university education	70 (25.4)
Employment status	
Employed	94 (34.1)
Unemployed	34 (12.3)
Retired	148 (53.6)
Marital status	
Unmarried	45 (16.3)
Married	192 (69.6)
Divorced	10 (3.6)
Widowed	29 (10.5)
Previous anesthesia experience	
Yes	227 (82.2)
No	49 (17.8)
Previous regional anesthesia experience	
Yes	98 (35.5)
No	178 (64.5)

Overall, 65.9% (n = 182) of participants preferred GA, whereas 34.1% (n = 94) preferred RA.

Factors associated with anesthetic preference

In bivariate analysis, anesthetic preference differed significantly according to sex, age, surgical specialty, educational level, employment status, and previous RA experience (all p < 0.001). RA was preferred by 46.2% (n = 74) of men compared with 17.2% (n = 20) of women. Patients preferring RA were older than those preferring GA (median age: 72.0 versus 61.0 years; p < 0.001). Among patients with previous RA experience, 48.0% (n = 47) preferred RA, compared with 26.4% (n = 47) of those without previous RA experience. Marital status (p = 0.061) and previous anesthesia of any type (p = 0.059) were not significantly associated with anesthetic preference. The bivariate comparisons are presented in Table 2.

**Table 2:** Association of participant characteristics with preference for general or regional anesthesia. Values are reported as counts (n) and percentages (%) unless otherwise indicated. A hyphen (-) indicates not applicable. Percentages are calculated within each participant characteristic category. Age is reported as median (IQR). P-values were calculated using Pearson's chi-square test for categorical variables and the Mann-Whitney U test for age. Bold values indicate statistical significance (p < 0.05). *Mann-Whitney U = 5917.0, Z = -4.198, and p < 0.001. IQR: interquartile range

Characteristics	General anesthesia, n (%)	Regional anesthesia, n (%)	P-value
Sex			
Male	86 (53.8)	74 (46.2)	-
Female	96 (82.8)	20 (17.2)	-
Age, years	61.0 (45.0-74.0)	72.0 (59.0-77.0)	<0.001*
Surgical specialty			<0.001
Orthopedics	61 (61.6)	38 (38.4)	-
General surgery	52 (85.2)	9 (14.8)	-
Urology	39 (45.9)	46 (54.1)	-
Obstetrics/gynecology	30 (96.8)	1 (3.2)	-
Educational level			<0.001
Primary education	47 (48.5)	50 (51.5)	-
Secondary education	51 (75.0)	17 (25.0)	-
High school	26 (63.4)	15 (36.6)	-
Higher/university education	58 (82.9)	12 (17.1)	-
Employment status			<0.001
Employed	81 (86.2)	13 (13.8)	-
Unemployed	23 (67.6)	11 (32.4)	-
Retired	78 (52.7)	70 (47.3)	-
Marital status			0.061
Single	25 (55.6)	20 (44.4)	-
Married	128 (66.7)	64 (33.3)	-
Divorced	10 (100.0)	0 (0.0)	-
Widowed	19 (65.5)	10 (34.5)	-
Previous anesthesia experience			0.059
Yes	144 (63.4)	83 (36.6)	-
No	38 (77.6)	11 (22.4)	-
Previous regional anesthesia experience			<0.001
Yes	51 (52.0)	47 (48.0)	-
No	131 (73.6)	47 (26.4)	-

In multivariable analysis, male sex was independently associated with higher odds of RA preference (OR = 7.12; 95% CI: 3.13-16.22; p < 0.001), as was previous RA experience (OR = 3.50; 95% CI: 1.70-7.19; p = 0.001). Surgical specialty (p = 0.005), educational level (p = 0.008), and employment status (p = 0.031) were also independently associated with anesthetic preference overall. Compared with orthopedics, patients undergoing general surgery (OR = 0.30; 95% CI: 0.10-0.90; p = 0.033) and obstetric/gynecologic surgery (OR = 0.09; 95% CI: 0.01-0.81; p = 0.032) had lower odds of preferring RA. However, this estimate should be interpreted cautiously because only one of the 31 participants in the obstetrics/gynecology subgroup preferred RA. Age was not independently associated with anesthetic preference after adjustment (OR = 0.99; 95% CI: 0.96-1.03; p = 0.728). No evidence of problematic multicollinearity was identified (all VIFs < 5), and the linearity-in-the-logit assumption for age was not violated (Box-Tidwell p = 0.086). The Hosmer-Lemeshow test was statistically significant (p = 0.006), indicating that model calibration was not optimal; therefore, the multivariable estimates should be interpreted with appropriate caution. Full results of the multivariable model are presented in Table 3.

**Table 3:** Multivariable logistic regression analysis of factors associated with preference for regional anesthesia. The dependent variable was preference for regional anesthesia (regional anesthesia = 1; general anesthesia = 0). ORs were adjusted for sex, age, surgical specialty, educational level, previous regional anesthesia experience, and employment status. Reference categories: female sex, orthopedics, higher/university education, no previous regional anesthesia experience, and retired employment status. A hyphen (-) indicates not applicable. Bold values indicate statistical significance (p < 0.05). OR, odds ratio; CI, confidence interval

Variable	OR	95% CI	P-value
Sex			
Female	1.00 (Reference)	-	-
Male	7.12	3.13-16.22	<0.001
Age, years	0.99	0.96-1.03	0.728
Surgical specialty			
Orthopedics	1.00 (Reference)	-	-
General surgery	0.30	0.10-0.90	0.033
Urology	1.37	0.61-3.11	0.446
Obstetrics/gynecology	0.09	0.01-0.81	0.032
Educational level			
Higher/university education	1.00 (Reference)	-	-
Primary education	0.35	0.08-1.56	0.168
Secondary education	0.17	0.04-0.68	0.012
High school	1.20	0.36-3.98	0.765
Previous regional anesthesia experience			
No	1.00 (Reference)	-	-
Yes	3.50	1.70-7.19	0.001
Employment status			
Retired	1.00 (Reference)	-	-
Unemployed	0.33	0.08-1.45	0.143
Employed	0.18	0.05-0.64	0.008

Patient-reported concerns regarding regional anesthesia

Among the 182 participants who preferred GA, the most frequently reported concerns regarding RA were hearing events during surgery (90.1%, n = 164), seeing the procedure (77.5%, n = 141), and experiencing intraoperative pain (73.1%, n = 133). Concern about the spinal injection was reported by 51.1% (n = 93). Concerns about permanent paralysis (40.7%, n = 74), back injury (40.1%, n = 73), and the loss of bodily control (39.6%, n = 72) were less frequent. Postoperative nausea or vomiting, postoperative headache, needles, and being unclothed were each reported by fewer than one-third of participants. Participants could report more than one concern. The complete distribution is presented in Table 4.

**Table 4:** Patient-reported concerns regarding regional anesthesia among participants preferring general anesthesia (n = 182). Values are reported as counts (n) and percentages (%). Participants could report more than one concern; therefore, percentages do not sum to 100%.

Concerns regarding regional anesthesia	n (%)
Hearing events during surgery	164 (90.1)
Seeing the procedure	141 (77.5)
Experiencing pain during surgery	133 (73.1)
Spinal injection	93 (51.1)
Permanent paralysis	74 (40.7)
Back injury	73 (40.1)
Loss of bodily control	72 (39.6)
Postoperative nausea/vomiting	52 (28.6)
Postoperative headache	43 (23.6)
Needles	42 (23.1)
Being unclothed	33 (18.1)

Information sources and desire for further information

Information-related items were completed by 202 of the 276 participants (73.2%). The remaining 74 participants did not provide responses to these items and were not included in the corresponding analyses; no imputation was performed. Among the 202 respondents, 31.2% (n = 63) reported having heard that RA could cause complications. Among these participants (n = 63), relatives were the most frequently reported source of information (66.7%, n = 42), followed by other patients (25.4%, n = 16) and personal beliefs or thoughts (7.9%, n = 5). Of the 202 respondents, 95.5% (n = 193) indicated that they wanted their anesthesiologist to address their questions regarding RA.

## Discussion

In this cross-sectional study, approximately two-thirds of patients preferred GA despite RA being a clinically viable option. Male sex and previous RA experience showed strong independent associations with RA preference, while surgical specialty, educational level, and employment status were also independently associated with anesthetic preference. Importantly, among patients preferring GA, the predominant concerns centered on hearing or seeing events during surgery and experiencing intraoperative pain rather than rare neurological complications. Almost all respondents to the relevant question wanted their anesthesiologist to address their questions regarding RA.

The predominance of GA preference is consistent with previous studies showing that many patients favor GA even when RA is feasible [2,3,6]. Capdevila et al. identified patient choice, fear of the alternative technique, anxiety or stress, expected recovery, and perceived efficacy among factors influencing the selection of spinal anesthesia versus GA [2]. Lavado et al. similarly reported that patients preferring GA perceived it as safer and expressed greater concern about remaining awake and experiencing pain [6]. These findings suggest that anesthetic preference reflects not only clinical considerations but also patients' expectations of the perioperative experience.

Sex showed one of the strongest independent associations in our analysis, with men having substantially higher odds of preferring RA. Dove et al. similarly reported a greater preference for GA among women and found that sex and previous anesthetic experience influenced preference [3]. Sex-related differences in the anxiety and fear of anesthesia have also been reported in a Greek surgical population [9]. Because anxiety and other psychological characteristics were not measured in the present study, however, the mechanisms underlying this association cannot be determined. Moreover, the relatively wide confidence interval around the effect estimate indicates uncertainty regarding the magnitude of this association, and residual confounding cannot be excluded. Therefore, the observed association should not be interpreted as evidence of an inherent sex-based difference in anesthetic preference.

Previous RA experience was also strongly associated with preference, with participants who had previously undergone RA having approximately 3.5-fold higher odds of preferring it. This finding is consistent with previous evidence showing an association between recent RA experience and subsequent preference [3]. Previous exposure should not necessarily be interpreted as a positive experience, however. Lavado et al. found that some patients previously exposed to neuraxial anesthesia did not wish to undergo it again [6]. In our study, previous anesthesia of any type was not significantly associated with anesthetic preference, whereas previous RA experience showed a strong independent association with RA preference. This distinction suggests that familiarity with the specific anesthetic technique, rather than previous exposure to anesthesia in general, may be more relevant to subsequent preference.

The pattern of patient-reported concerns is particularly relevant. Hearing events during surgery, seeing the procedure, and experiencing intraoperative pain were reported more frequently than permanent paralysis or back injury. Earlier studies have identified pain, needles, neurological injury, and paralysis as barriers to RA [4,10]. Dove et al. reported that concerns about hearing or seeing surgery could exceed concerns about anesthetic complications [3], while Lavado et al. found greater concern about remaining awake and experiencing pain among patients preferring GA [6]. Our findings therefore indicate that concerns related to the anticipated sensory experience of surgery while conscious were more frequently reported among patients preferring GA than concerns about rare neurological complications. These concerns were reported in association with GA preference and should not be interpreted as definitive causal reasons for that preference, as the cross-sectional design does not permit causal inference. Patients' perceptions of RA may also differ according to the specific regional technique being considered and whether sedation is offered, factors that may influence how concerns about remaining conscious or experiencing the surgical environment are perceived.

This distinction has practical implications for preoperative counseling. Discussions about RA appropriately include its benefits, alternatives, adverse effects, and potential complications. However, patients may also benefit from explicit information about what they are likely to hear, see, or feel during surgery and whether sedation can be provided when clinically appropriate. Such communication may help establish realistic expectations and support informed choice rather than persuade patients toward a particular anesthetic technique.

The demand for professional information reinforces this point. Among the 202 respondents to the information-related items, approximately one-third had heard that RA could cause complications, with relatives and other patients representing the main sources of this information. In contrast, 95.5% (193/202) wanted their anesthesiologist to address their questions regarding RA. Previous Greek data similarly demonstrated substantial preoperative anxiety and a strong desire to discuss anesthesia-related fears with the anesthesiologist [9]. Gaps in patient knowledge regarding RA have also been reported [5]. Multimedia preoperative information may reduce anxiety in patients undergoing RA [11], while educational interventions have been associated with improved knowledge and changes in anesthetic preference [12,13].

More recent evidence emphasizes that the quality and individualization of information may be as important as its quantity. A 2025 systematic review concluded that the amount, source, and quality of preoperative information may influence fear and anxiety and emphasized person-centered communication tailored to patients' needs [14]. Similarly, the introduction of a dedicated RA information leaflet improved patients' understanding of risks and benefits and satisfaction with the consent process [15]. Education should therefore facilitate informed choice rather than simply increase the uptake of RA.

Patient involvement in anesthetic decisions is also important. Hwang et al. found that most patients preferred an active or collaborative role in selecting their anesthetic technique and reported high satisfaction after participating in the decision [16]. More recent research has similarly demonstrated interest among surgical patients in patient-led or shared anesthetic decision-making [17]. A recent cross-sectional study further demonstrated the complexity of shared decision-making in anesthesia, showing differences in perceived involvement between patients and clinicians and changes in anesthetic preference following pre-anesthetic evaluation [7]. These findings are consistent with the high demand for professional information observed in our cohort.

The Greek context also deserves consideration. Earlier Greek research found that patients declining RA expressed more anesthesia-related fears and questions, while older age was associated with consent to RA; previous anesthesia of any type was not associated with the decision regarding RA [18]. Similarly, previous anesthesia of any type was not significantly associated with preference in our study, whereas previous RA experience remained independently associated with RA preference. Subsequent Greek data demonstrated substantial preoperative anxiety and a strong desire to discuss anesthesia-related fears with the anesthesiologist [9]. Although RA is well established in Greek anesthetic practice and continuing efforts have focused on regional anesthesia education and training, our findings suggest that patient-related concerns remain relevant [19,20].

Surgical specialty, educational level, and employment status were independently associated with anesthetic preference, but these findings require cautious interpretation. They may partly reflect differences in surgical procedures, demographic composition, socioeconomic characteristics, or previous healthcare exposure. Age differed significantly between preference groups in bivariate analysis but was no longer independently associated with preference after adjustment. Anesthetic preference should therefore be viewed as multifactorial and context-dependent rather than attributable to a single demographic characteristic.

Strengths and limitations

A strength of this study is that questionnaires were completed before the preoperative anesthetic consultation, capturing patients' baseline preferences and concerns before individualized counseling. The study also examined specific patient-reported concerns regarding RA across several surgical specialties.

This study has several limitations. Its single-center, cross-sectional design limits causal inference and generalizability, while preferences assessed before formal consultation represent initial rather than final anesthetic decisions. Neuraxial anesthesia and peripheral nerve blocks were grouped as RA, despite potentially different patient perceptions, and sedation and combined GA/RA approaches were not separately incorporated into the preference framework. Residual confounding cannot be excluded, and the significant Hosmer-Lemeshow test indicated suboptimal model calibration. The sample-size calculation was not designed specifically for multivariable logistic regression; the final model included 11 parameters for 94 RA preference events (approximately 8.5 events per parameter), warranting caution particularly for estimates with wide confidence intervals.

Information-related items were completed by a smaller subset (202/276). Individual surgical procedures, the specific RA technique previously received, and patients' satisfaction with that experience were not recorded, and the predefined concerns did not allow free-text responses. Finally, although the study-specific questionnaire was literature-informed, reviewed by experienced anesthesiologists, and pilot-tested for clarity and comprehensibility, it did not undergo formal psychometric validation.

## Conclusions

Approximately two-thirds of elective surgical patients preferred GA despite RA being a clinically viable option. Male sex, previous RA experience, surgical specialty, educational level, and employment status were independently associated with anesthetic preference. Among patients preferring GA, the leading concerns centered on hearing or seeing events during surgery and experiencing intraoperative pain rather than rare neurological complications, suggesting that expectations of the intraoperative experience may play an important role in anesthetic preference. The strong demand for professional information highlights the importance of individualized preoperative counseling that addresses not only the risks and benefits of RA but also patients' practical concerns and expectations. Such communication may support informed, patient-centered discussion and decision-making without necessarily altering patients' anesthetic preferences. Future multicenter studies should evaluate whether targeted counseling addressing these specific concerns influences subsequent anesthetic decisions.

## Figures and Tables

**Figure 1:**
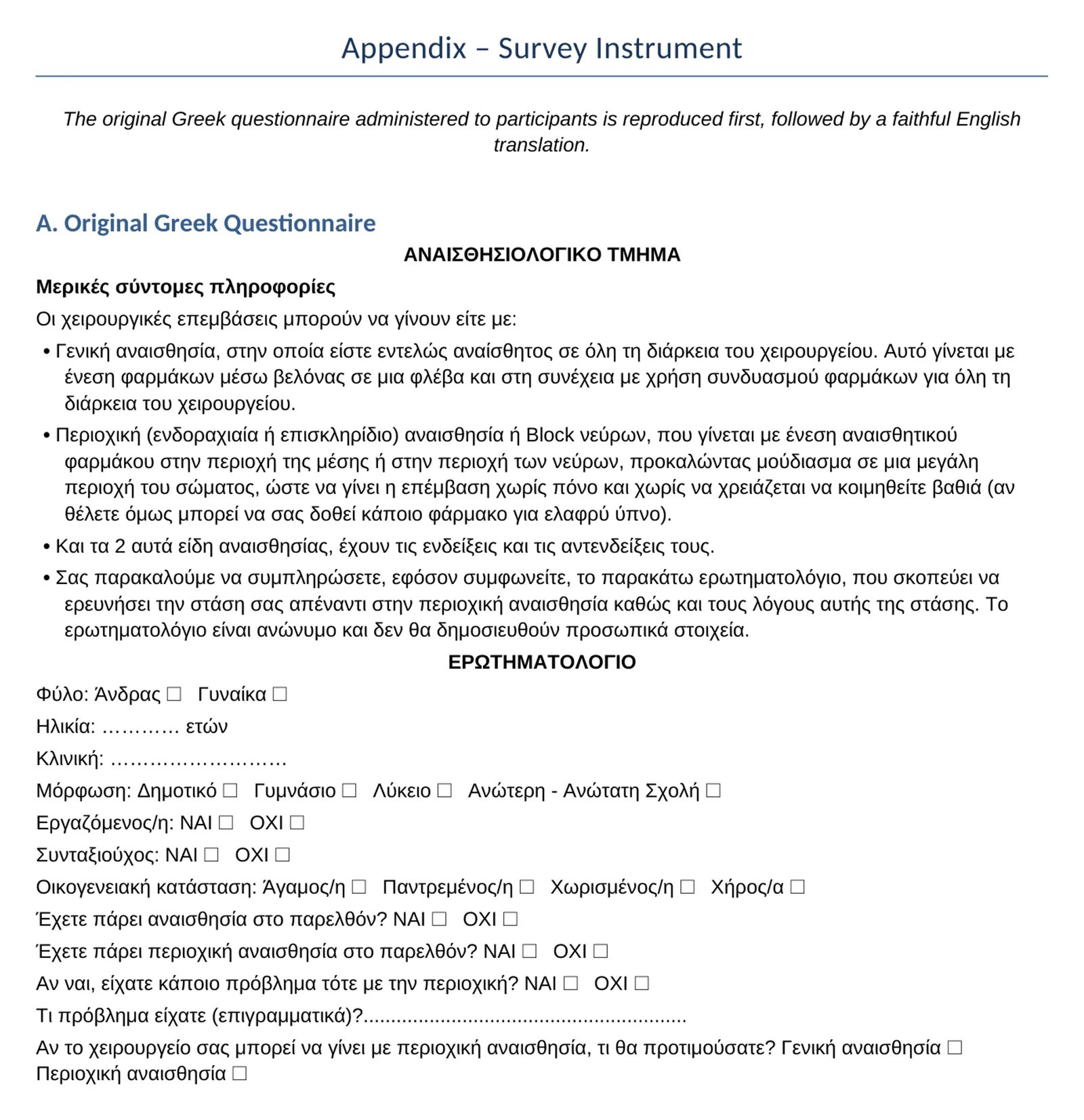
Survey on patient preferences and concerns regarding regional anesthesia. This is part 1 of the original Greek questionnaire administered to study participants.

**Figure 2:**
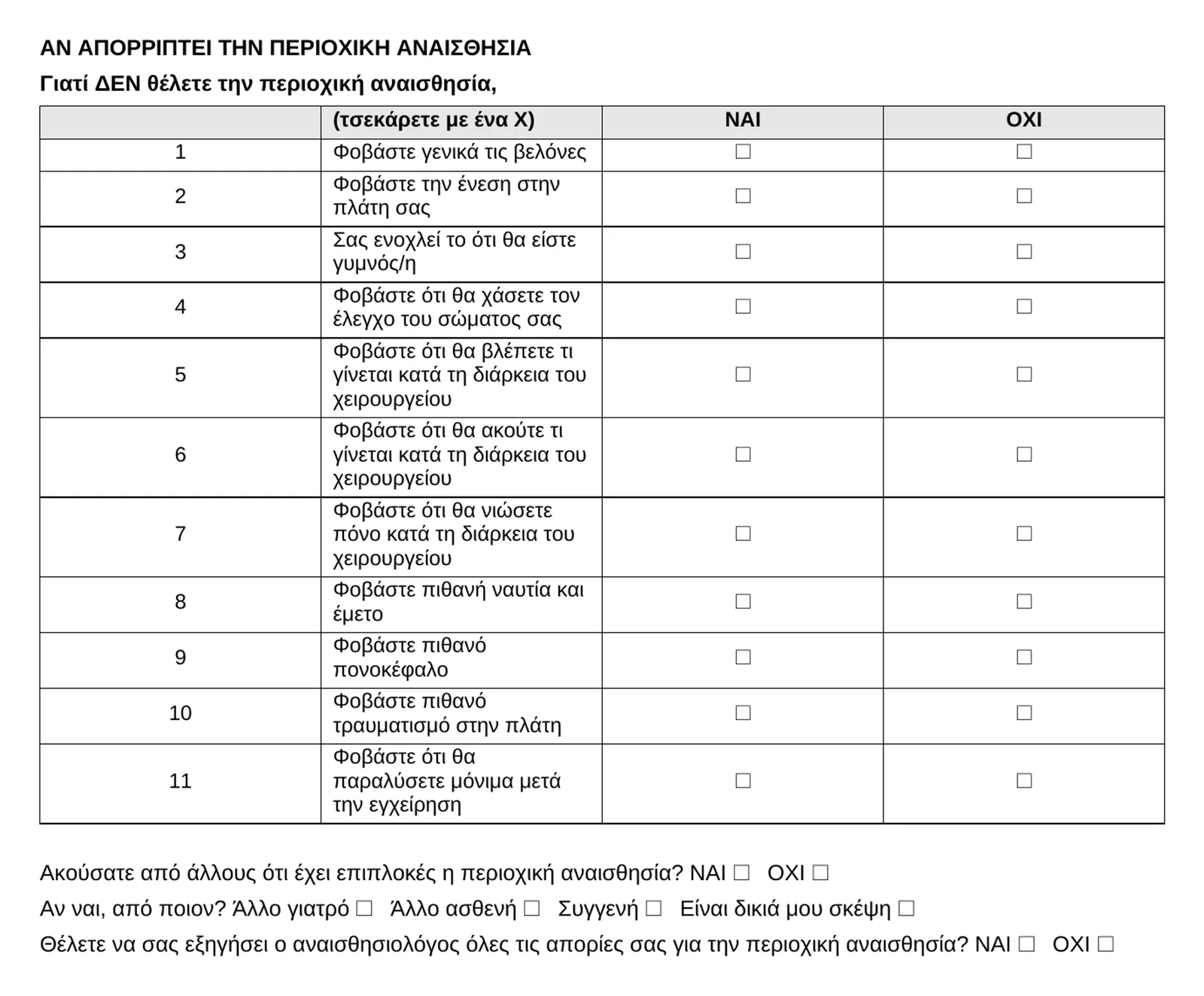
Survey on patient preferences and concerns regarding regional anesthesia. This is part 2 of the original Greek questionnaire administered to study participants.

**Figure 3:**
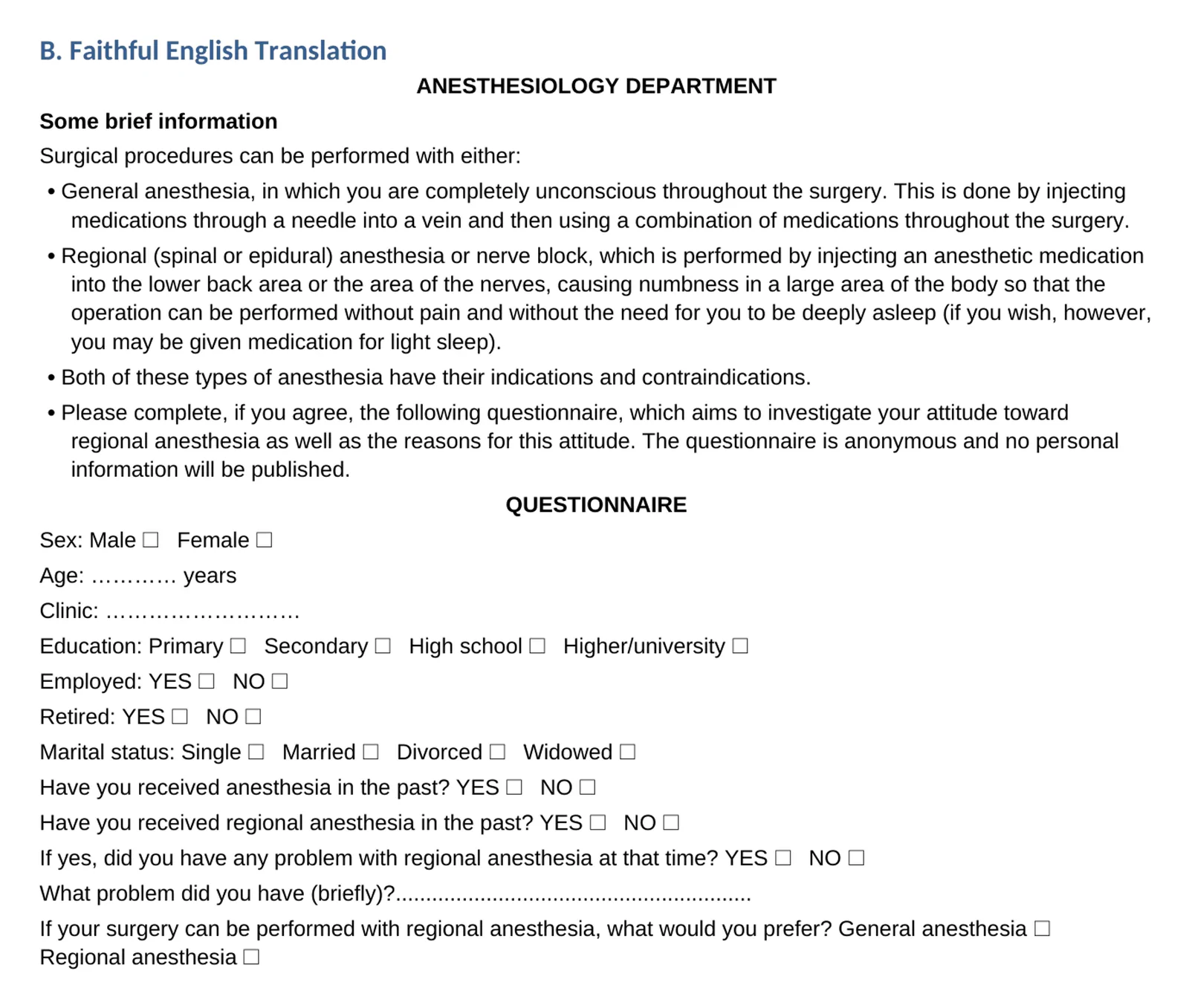
Survey on patient preferences and concerns regarding regional anesthesia. This is the English translation of part 1 of the original Greek questionnaire administered to study participants.

**Figure 4:**
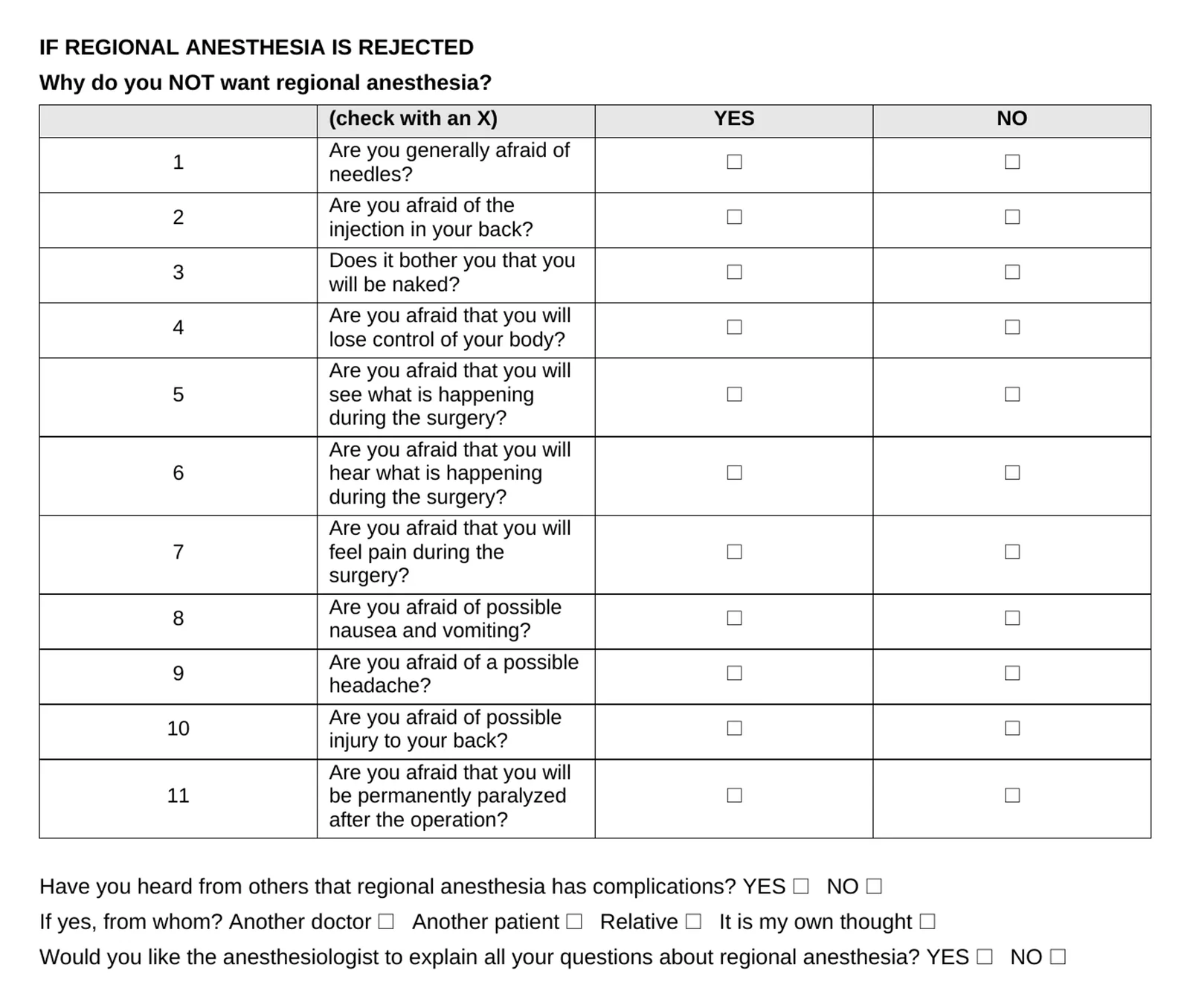
Survey on patient preferences and concerns regarding regional anesthesia. This is the English translation of part 2 of the original Greek questionnaire administered to study participants.

## Appendices

Figures 1-4 show he complete study-specific questionnaire administered to participants, including the original Greek version and its English translation.
